# Computational Methods for Liver Vessel Segmentation in Medical Imaging: A Review

**DOI:** 10.3390/s21062027

**Published:** 2021-03-12

**Authors:** Marcin Ciecholewski, Michał Kassjański

**Affiliations:** Department of Geoinformatics, Faculty of Electronics, Telecommunication and Informatics, Gdańsk University of Technology, 80-233 Gdańsk, Poland; kassjanski@gmail.com

**Keywords:** liver vessels, segmentation, liver vessel segmentation, vascular segmentation, segmentation methods, medical imaging, review

## Abstract

The segmentation of liver blood vessels is of major importance as it is essential for formulating diagnoses, planning and delivering treatments, as well as evaluating the results of clinical procedures. Different imaging techniques are available for application in clinical practice, so the segmentation methods should take into account the characteristics of the imaging technique. Based on the literature, this review paper presents the most advanced and effective methods of liver vessel segmentation, as well as their performance according to the metrics used. This paper includes results available for four imaging methods, namely: computed tomography (CT), computed tomography angiography (CTA), magnetic resonance (MR), and ultrasonography (USG). The publicly available datasets used in research are also presented. This paper may help researchers gain better insight into the available materials and methods, making it easier to develop new, more effective solutions, as well as to improve existing approaches. This article analyzes in detail various segmentation methods, which can be divided into three groups: active contours, tracking-based, and machine learning techniques. For each group of methods, their theoretical and practical characteristics are discussed, and the pros and cons are highlighted. The most advanced and promising approaches are also suggested. However, we conclude that liver vasculature segmentation is still an open problem, because of the various deficiencies and constraints researchers need to address and try to eliminate from the solutions used.

## 1. Introduction

Two parts can be distinguished in the liver vein system: hepatic veins and the portal vein. They have a very complex, tree-like structure. According to anatomy, venous blood flows through the portal vein, into liver, and then flows into the inferior vena cava through the hepatic veins. Hepatocellular carcinoma constitutes one of the most deadly cancers in the world [1]. Computer-assisted liver surgery (e.g., ablation and embolization) allows the clinical treatment of unresected liver tumors. Prior to this treatment, it is very important for physicians to have information about the liver contour and about its venous system. It is used for preoperative planning and intraoperative navigation. Liver vessels that feed neoplasms should be accurately located during liver embolization surgery. Knowing the detailed location of the tumor between liver vessels and information such as the diameter of vessels can determine the outcome of the ablation. New technologies are constantly being introduced into clinical practice to improve the ability to visualize blood vessels, including those of liver. They include: computed tomography (CT), computed tomography angiography (CTA), magnetic resonance (MR), and ultrasonography (USG).

**Figure 1:**
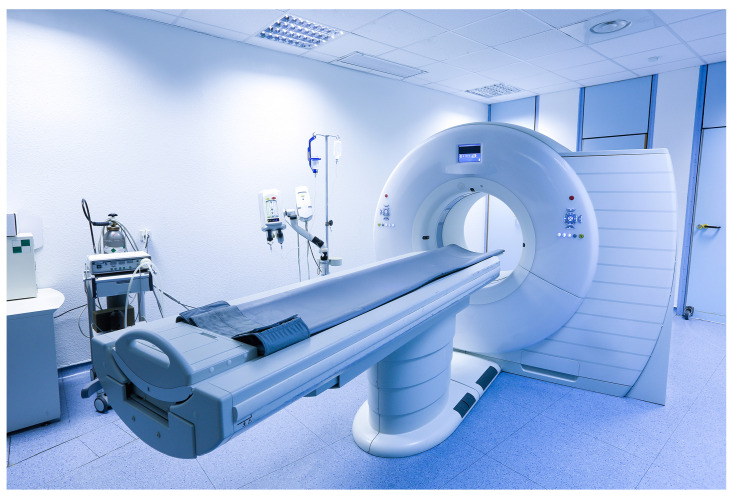
A CT scanner suitable for imaging the abdominal cavity and liver (courtesy of @ zlikovec/Depositphotos.com).

**Figure 2:**
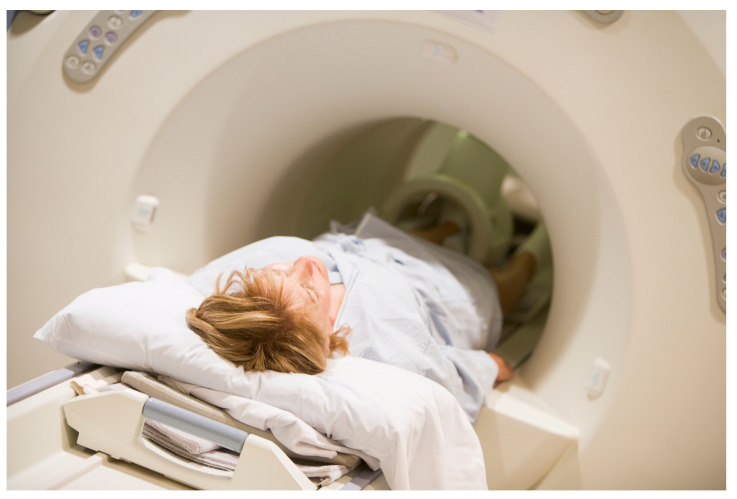
A CT examination of a patient to scan the abdominal cavity and liver (courtesy of @ monkeybusiness/Depositphotos.com).

**Figure 3:**
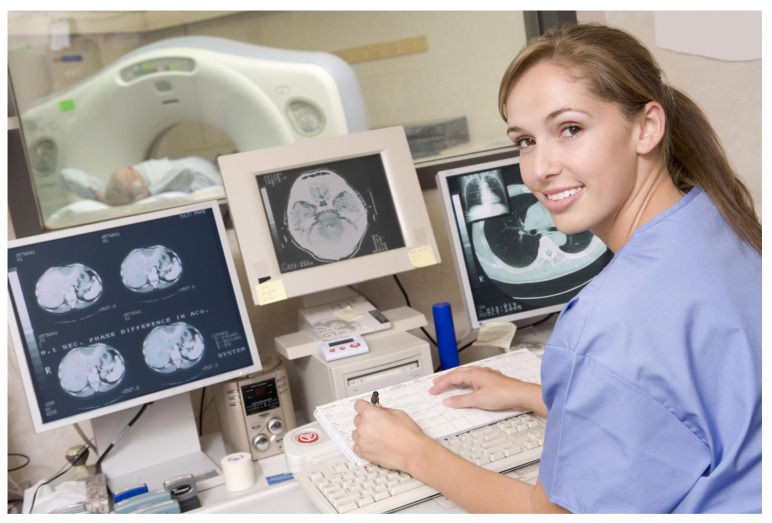
A nurse during a CT examination with the operating software visible (courtesy of @ monkeybusiness/Depositphotos.com). The monitor on the left displays example slices of the abdominal cavity and liver. Liver slices are shown in more detail in Figure 4.

**Figure 4:**
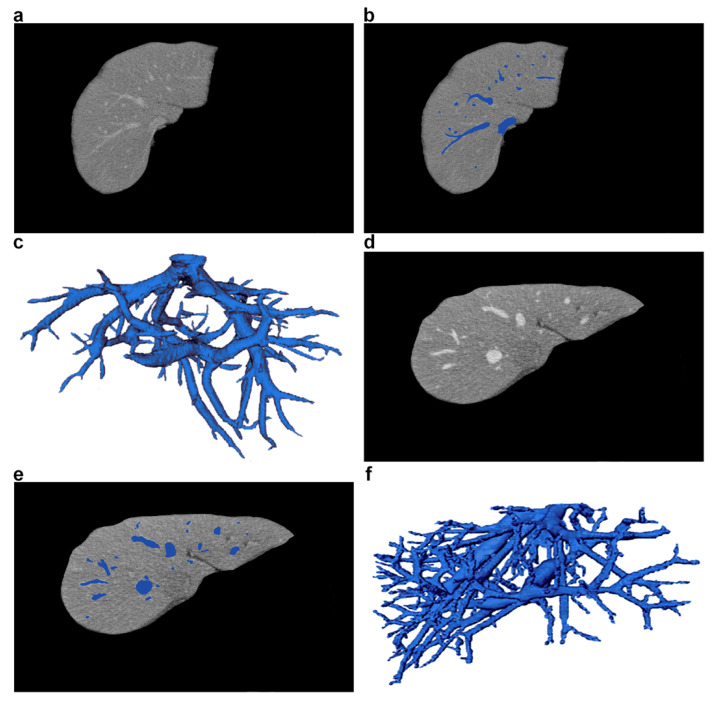
Sample CT images of liver and its blood vessels, taken from two different patients. (**a**,**d**) CT liver slices with vessels (axial view). (**b**,**e**) Vessel segmentation marked in blue. (**c**,**f**) 3D visualization of liver vessel trees.

Figure 1 shows an example of a CT scanner, and Figure 2 shows a CT examination of the abdominal cavity and liver. Figure 3 shows a nurse monitoring patient during a CT scan. Figure 4 shows sample CT images of liver and a 3D visualization of its blood vessels.

Manual liver vessel segmentation is time consuming and tedious and often becomes impossible if the number of medical images is high. In addition, its results may vary, as they depend on the operators’ experience and skill.

Automated and semi-automated image processing tools can reduce the number of manual interactions and greatly simplify the work of physicians, which is why they are attracting the increasing attention of researchers, as proven by the large number of articles published on this subject. There are many studies of blood vessel segmentation in the literature, including review papers in this field [2,3,4,5,6]. However, due to the intensive progress in research, new review papers are needed to update the state-of-the-art and examine the results obtained in detail.

**Figure 5:**
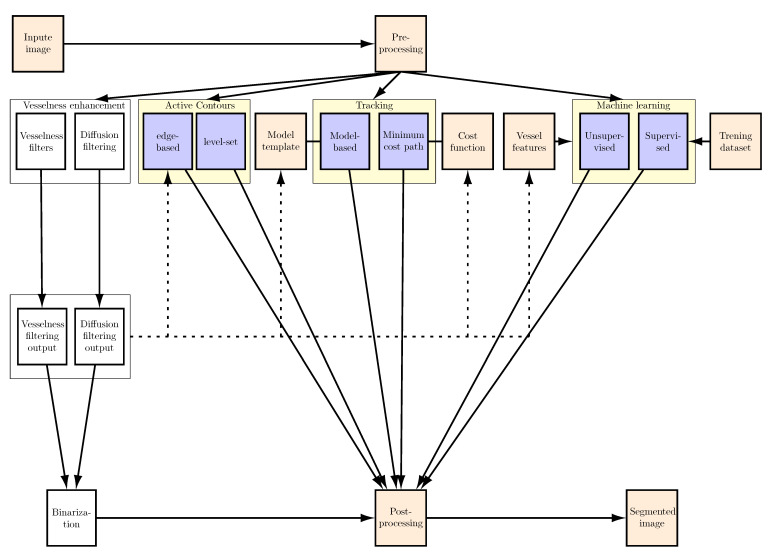
Diagram showing methods used to segment liver vessels, based on a literature review. These methods are highlighted in yellow. Pre-processing is intended to enhance vessels and is used by segmentation methods (shown with dotted lines). Post-processing improves the segmentation results.

This paper is provides a systematic review of current methods of liver vessel segmentation and classification, based on the literature. Each group of methods is briefly presented in terms of its ability to segment images in general and then liver vessels in particular. The pros and cons of the approaches used are also described for each group of methods. What is also presented are the metrics used to evaluate segmentation results and publicly available datasets. The paper also discusses possible future directions of research on liver vessel segmentation. Figure 5 presents the classification of the approaches, based on papers referred to in this review. Table 1, in turn, summarizes articles including the year of publication and the imaging technique.

During the literature review, four databases (Google Scholar, IEEE-Explore, Scopus, Springer) were searched using the following search terms: ((liver vessel segmentation) and (hepatic vessel segmentation) and (liver vessel segmentation deep learning) and (CT or CTA or MR or USG)). The analyzed period covered results from 2010–2021. The following criteria were adopted to reject articles: (a) articles not written in English; (b) articles not developing any segmentation method; (c) research not related to liver vessel segmentation; (d) duplicates of articles from the same research project; (e) articles in which tests and validations of segmentation methods were not conducted. As a result, 31 publications were selected and are shown in Table 1.

The remainder of this paper is organized as follows. Section 2 describes the division of liver vessel segmentation methods into different groups according to the literature review. Section 3 presents measures of segmentation performance, and Section 4 lists publicly available datasets that were used for research. The next three sections describe state-of-the-art approaches based on active contour methods (Section 5), tracking methods (Section 6), and machine learning (Section 7). The last section contains challenges and conclusions.

## 2. Methods of Segmenting Liver Blood Vessels

At the pre-processing stage, input images are filtered to eliminate noise, enhance contrast, and extract features (Figure 5). Different pre-processing methods are employed because the imaging techniques (i.e., CT, CTA, MR, USG, according to Table 1) and equipment used produce images of different resolution, contrast, and noise in them. A detailed literature review concerning the different pre-processing methods was presented in [39].

Methods for segmenting liver blood vessels can be divided into the following groups:

- Vessel enhancement approaches
- Active contour methods
- Tracking methods
- Machine learning approaches

Vessel enhancement approaches improve the contrast of vessels and make them easier to extract from the background of the image, for example by applying thresholding. However, as Figure 5 shows, vessel contrast enhancement also constitutes the initial stage used by other methods to produce the final segmentation. There are comprehensive reviews of the literature on enhancement methods [3,40,41], so these methods are not described here. The last step in segmentation is post-processing, which has a supporting role and is intended to, e.g., remove noise and minor artifacts that do not correspond to vessels, as well as to connect vessel sections if they are incomplete as a result of the segmentations done.

## 3. Segmentation Metrics

Evaluating completed segmentations makes it possible to compare the performance of methods and also to improve them and/or develop new, better solutions. This section presents segmentation performance measures implemented in all reviewed studies. Table 2 summarizes segmentation performance measures on the basis of thirty one articles. However, this paper rather rarely deals with the reliability and consistency of the results of individual studies, based on a review of the literature.

## 4. Publicly Available Evaluation Datasets

Table 3 lists five publicly available datasets containing images for segmenting and producing 3D visualizations of liver blood vessels. Three datasets are used for research according to a current review of the literature, namely:

- Segmentation of the Liver Competition 2007 (SLIVER07) [51]
- 3D Image Reconstruction for Comparison of Algorithm Database (3D-IRCADb) [53]
- Vascular Synthesizer [54]

The dataset called Medical Segmentation Decathlon (MSD) [55] has not been included in research results yet as it has only recently been published, but it contains vascular ground-truths. The second dataset in which vascular ground-truth masks have been made available is 3D-IRCADb [53]. The Combined (CT-MR) Healthy Abdominal Organ Segmentation—CHAOS challenge database [56] is available as a set of CT and MR images of liver and its blood vessels. It has only been published recently and has not yet been used in research.

**Table 3:** Publicly available evaluation datasets. SLIVER07, Segmentation of the Liver Competition 2007; 3D-1RCADb, 3D Image Reconstruction for Comparison of Algorithm Database; MSD, Medical Segmentation Decathlon.

Name	Number of Images/Volumes
SLIVER07 [51]	30 CT
3D-1RCADb [53]	22 CT
(http://www.ircad.fr/research/3dircadb/)	
(accessed on 5 May 2019)	
MSD [55]	443 CT
(http://medicaldecathlon.com/)	
(accessed on 20 December 2020)	
CHAOS [56]	50 CT and 59 MR
(https://chaos.grand-challenge.org/Data/)	
(accessed on 20 December 2020)	
Vascular Synthesizer [54]	120 (3D synthetic data)

Unfortunately, manual segmentation is a tedious and time-consuming task. What is more, it requires much input from experts. Synthetic data, in turn, allow representing certain important characteristics of the vessel tree, such as its thickness, tortuousness, and brightness profile [54]. These characteristics are important for evaluating methods under development. In addition, synthetic data can easily be modified. However, despite these advantages, phantoms cannot replace clinical data, including, e.g., the high variability of vessel shape, which results from patients’ individual traits. This is why some studies use both real medical images and synthetic data for the best possible matching of the necessary parameters and obtaining reliable results. Unfortunately, there is no publicly available dataset for ultrasonographic (USG) images in Table 3. This is because such a dataset does not exist yet.

## 5. Active Contour Methods

The active contour model (ACM) is a method that allows adapting a deformed curve or curves to detect the boundaries of an object. The models developed include: (a) models with an explicit representation of the curve; (b) models with an implicit representation of the curve.

An example of models with an explicit curve representation are the edge-based methods [57,58]. Edge-based models use local edge information (e.g., gradient) to fit to the boundaries of the approximated shape in the image.

In the case of the implicit representation, the ACM is expressed using the level set method [59,60]. When the level set method is used, the object contour is defined by a zero level set. The contour adapts in successive iterative steps, which are established based on a partial differential equation (PDE) for the defined energy functional. Methods utilizing level sets cope much better with topological changes of contours than methods with an explicit representation of the curve (contour) and support, for example detecting boundaries of multiple objects. This class of methods seems to be suitable for segmenting complex vessel trees, including those with large variations in shape and size. These methods may be useful for segmenting vessels in images from healthy patients, as well as those showing vascular pathologies. However, the use of these methods in real-time applications is limited by their computational cost.

### 5.1. Edge-Based Method

Reference [10] presented an ACM with shape and size constraints on the cross-section of vessels. The proposed approach starts with the extraction of the vessel axis from the source 3D CTA data. Next, the vessel boundary is delineated on the vessel cross-section from this axis, and then, an ACM is applied to segment vessels under constrained movements.

### 5.2. Level-Set Method

Zeng et al. [7] used the ACM presented in [61], which utilizes boundary information to obtain the correct location of the blood vessels being approximated and region information to stop the contour from leaking beyond the boundaries of vessels. The ACM was used together with the K-means clustering method [62] to improve thick vessel extraction. The K-means clustering method was used to enable initializing the ACM.

In [8], an active contour method was proposed, which uses image information, namely regional intensity distribution and a calculated map of the probability of the occurrence of blood vessels, to segment both thick and thin vessels. This approach allows segmenting both the portal vein and the hepatic vein in such a way that these veins do not need to be separated from each other. What is more, connections between the portal and hepatic veins do not affect the performance of the method developed.

Lu et al. [9] presented a variational level set method for segmenting liver blood vessels in magnetic resonance (MR) images. This approach uses non-local robust statistics to reduce noise in MR images. The non-local robust statistics that allow vascular features to be represented are adaptively learned based on seed points marked by the user.

Shang et al. [11] developed the vascular active contour model (VAC) for segmenting blood vessel trees. The VAC model uses: data on the intensity distribution in the region of the vessel, as well as the multi-scale vascular vector field and curvatures related to tubular structures. The vascular vector field allows the active contour to evolve along its centerline into thin and weak vessels. The combination of a region-based active contour model with the vascular vector field allows segmenting large vessels and accurately detecting thin vessels at the same time.

A summary of the analyzed active contour methods is presented in Table 4.

## 6. Tracking Methods

Methods for tracking blood vessels should be initiated using a single seed point or a specified number of them, and then, subsequent points are found based on image-derived data. Seed points can be initiated manually or derived from image pre-processing methods. Vessel tracking algorithms can be useful for segmenting very branched vascular trees for which a certain number of seed points initiated separately for each branch must be used. Among the existing approaches, one can distinguish methods that track vessels according to the vascularization model, as well as minimum cost path methods (MCP), which determine the minimum path between two seed points based on image-derived metrics.

### 6.1. Model-Based

These methods use certain predefined 3D models, usually tubular. After the model is initialized, a new position is obtained in every subsequent iteration by finding the best match. The match is calculated based on specific image features (e.g., intensity, gradient) that are determined in the neighborhood of the current model position. Unfortunately, these approaches can identify incomplete blood vessels, which may be due to intensity inhomogeneity, noise, and also the presence of pathologies.

The approach described in [22] utilizes a 3D geometrical moment-based detector to determine the vessel center, diameter, and local direction. This step allows automatic tubular structure detection. Then, the detected structures are described as vessel presence probabilities and used as a local constraint in a graph cut segmentation [63]. These methods avoid the shrinking bias on elongated structures, which often appears when the graph cuts algorithm is used.

In [20], an approach was developed in which vessel skeletons are extracted using tubular structures. Segmentation was performed using the graph cuts method [63], which splits the image into two different sets, i.e., the vessel and the background. The proposed solution allows tubular structures to be grouped into complete trees and also interwoven trees to be separated. The use of structural information about tubular trees is also enabled to reduce segmentation errors such as under segmentation or leakage.

Sangsefidi et al. [15], in turn, presented a modified graph cuts algorithm. A conventional graph cuts algorithm uses the image gradient and does not have sufficient data to extract vessel boundaries, especially of vessels that are small and in low contrast regions. This is why the authors of [6] defined a balanced data term of graph cuts to improve the results of segmenting liver blood vessels. In [15], vessel centerlines were used to calculate the expression of a local data term from the image, which can then be used to balance the calculated total energy in regions with low contrast and small vessels.

The approach taken in [14] is to propose an intensity model based on kernel fuzzy C-means to extract intensity features of thick vessels. In addition, a centerline constraint and intensity model were used to determine the position and distance for identifying thin vessels. Then, the centerline constraint and intensity model were integrated into graph cuts to produce the final tree of liver blood vessels.

Guo et al. [12] first pre-processed CT images using a Hessian filter and an anisotropic filter to enhance the contrast of liver vessels and remove noise. In the next step, they used an improved three-dimensional graph cuts algorithm for the initial segmentation of vessels. Subsequently, vessel centerlines were determined using a thinning algorithm, and the connectivity of these lines was ensured. False branches were deleted.

In [13], blood vessels were segmented in several steps. First, the vesselness filter proposed by Sato et al. [64] was used for principal component extraction. This filter was used with three parameters to: (1) select the scale of the structures to be detected, (2) define the appropriate size of tubular structures, and (3) enhance contrast (i.e., remove non-uniform contrast). Then, the 3D model extracts vessels and extends centerlines. In the next step, vessels are connected to each other according to their anatomy. Then, a non-linear operator, called RORPO (ranking the orientation responses of path operators) [65], is applied to the partial vessel skeleton produced in the previous step. The RORPO operator allows the intensity of curvilinear structures to be maintained. In the next step, anisotropic diffusion filtering [66] is used, and the gradient magnitude is calculated. At the end, a three-dimensional vessel reconstruction is performed for each voxel [67].

Yang et al. [16] presented the segmentation of liver vessels, namely the portal vein (PV) and the hepatic vein (HV), based on identified seed points and threshold intervals, using the region growing method implemented in the Insight Toolkit (ITK) environment [68]. The region growing method begins the segmentation from the identified seed points, then finds adjacent voxels and adds them to voxels already identified if the former voxels are within the defined threshold intervals. Connected vessel branches are then identified by the connected component analysis method in ITK [68]. If the PV and HV are interconnected, the user can use a scalable 3D sphere to remove the voxels connecting them.

In [18], a method for extracting the liver venous tree was presented. First, potential vessel intersection points between portal and hepatic venous systems are extracted. Then, every neighborhood of a vessel intersection is modeled using a robust twin-line random sample consensus (RANSAC) shape detector. Yan et al. [18] found that the RANSAC method is insensitive to deformations in vessel masks and noise. The last step is to segment the venous tree based on results produced by the RANSAC method and physical constraints posed by Murray’s law [69], which are determined for the vessel radii at each bifurcation.

Chi et al. [19] proposed a liver vasculature segmentation method, which locally groups voxels into branchings and then globally groups different vessel systems using a multiple feature point voting mechanism. Vessel context-based voting allows blood vessels of liver to be identified and segmented using region-based features. The vessel context defines the context information of a voxel, related to certain vessel properties such as intensity, connectivity, direction, and saliency. Vessel context is updated after each successful grouping operation until all branches are matched.

### 6.2. Minimum Cost Path

This method allows the shortest path to be found between two selected points ($pt_{1}$ and $pt_{2}$) by minimizing the energy functional, which is influenced by the metric tensor *M* [70]. *M* here is defined based on selected image features such as intensity, gradient, or higher order derivatives. The $pt_{1}$ point is the only global minimum of the functional *M*. Energy is minimized by calculating the minimum action map, which is usually determined using the fast marching method (FMM) [71]. Then, the path between points $pt_{1}$ and $pt_{2}$ is obtained along with decreasing gradient values from the resulting map, where $pt_{2}$ is the starting one.

Zeng et al. [17] used optimal oriented flux and oriented flux antisymmetry [72,73]. They combined these two methods to detect blood vessels. Then, after vessel centerlines were automatically extracted, the FMM method was used together with graph cuts to perform the segmentation. The approach presented in [17], including the extraction of centerlines, allows small, thin, and overlapping vessels to be effectively segmented.

In [21], first the Hessian-based vesselness filter was used to allow vessels of a certain diameter to be enhanced. Then, iterative ridge-oriented region growing, whose implementation was based on the FMM method, was applied. Skeleton-based post-processing was also used to interactively correct vessel segmentation errors.

The approach proposed in [23] is executed in two steps. In the first step, the boundaries of larger vessels are extracted using the graph cuts method. This way, larger vessels are detected globally, based on estimates of certain parameters (e.g., intensity contrast, noise level). Then, smaller vessel branches are segmented by the vessel tracking method based on results obtained using the medialness filter [23]. The medialness filter presented in [23] uses the expected intensity profile and the assumption of the circularity of vessels processed. Smaller vessels are tracked with the minimal path detection method, which is executed on a discrete grid, whereas the costs of graph edges are computed from the results obtained using the medialness filter.

Table 5 summarizes the segmentation results obtained using tracking methods.

## 7. Machine Learning Methods

Two groups of machine learning methods can be distinguished: supervised and unsupervised. Supervised learning uses a dataset that contains the expected answer. For segmentation, these are ready reference examples that must be provided to train the learning model. In unsupervised learning, on the contrary, no answers are provided, only a dataset. This approach is useful if no public datasets are available that would include, e.g., examples of correct segmentations, or if such data are not used.

### 7.1. Unsupervised

Unsupervised learning models use certain special characteristics from the statistical distribution, based on input data. These models learn to label every image without knowing ground-truth labels. The lack of available reference segmentations justifies the use of such models. However, the results obtained are generally poorer than from supervised methods. These models conduct the segmentation based on indeterminate image features such as the local intensity and gradient.

Goceri et al. [33] applied k-means clustering to the initial segmentation of blood vessels. Then, certain morphological operations were executed to improve the results. The method used can be adjusted to different brightness distributions in the image by selecting the appropriate morphological operators while using the k-means method, which operates automatically.

The authors of [37] proposed a method of region growing in which pixels are included based on a defined range of intensity values. The extreme values of the interval being determined are obtained by matching with Gaussian functions and using the Gaussian mixture model (GMM). This method can be used to segment liver blood vessels if the pixel brightness distribution in the image is close to Gaussian. The portal and the hepatic veins are separated using certain geometric features such as their size and connectivity.

A combination of four 3D vascular filters, including Sato [64], Frangi [74], offset medialness [75], and strain energy [76], was introduced in [35] to extract vessel features. Then, the extreme learning machine (ELM) method was used to recognize liver vessels and extract them from the background.

Wang et al. [36] presented the application of a multi-scale Hessian-based vesselness filter to enhance vessels of a defined diameter. Then, they used the Bayes classifier to identify the vessels. If there was no connection between vessels, two methods of connecting them were proposed. The first method uses a directional morphological operator to dilate sections of liver blood vessels along their centerline directions. This is to enable restoring connections between separated vessels. The second method checks connections between vessel sections and restores connectivity between them and vessel branches (e.g., to ensure the connection of bifurcations).

The authors of [38] used ant colony optimization, a metaheuristic, to find the best matching liver vessel tree using determined paths representing vessels and belonging to a predetermined graph. The potential paths based on which the liver vessel model is to be generated are produced using a cost path algorithm, which allows a graph to be created. This graph consists of all candidate vessel bifurcation locations as vertices and candidate vessel segments as edges.

Table 6 summarizes the segmentation results obtained using unsupervised learning methods.

### 7.2. Supervised

To segment blood vessels, certain features (e.g., intensity, gradient) are extracted from training images. Then, the machine learning model is trained using these features and the corresponding labels, which are obtained from ground-truth segmentations. After the training phase, this model can be used to segment liver blood vessels in test images.

The convolutional neural network (CNN) is a synthetic neural network with a feed-forward mechanism, in which the communication between neurones is inspired by the human visual cortex. In liver blood vessel segmentation, a CNN can be used to learn from consistent patterns from a training set consisting of reference images with annotations, and then, the CNN should allow predicting images from a test set. A CNN can also be utilized directly to segment vessels thanks to using fully connected layers. CNNs can model intensity patterns of objects having very different and variable appearance. During the training phase of a CNN, the convolutional layers of the network automatically generate features and combine them into hierarchical predictive models.

Artificial neural networks (ANNs) for deep learning can have 10+ layers or many more. Models that have as many as 100+ layers have been created [77,78]. Deep convolutional networks enable gradual filtering of different parts of training data and can fine-tune features that are important in the discrimination process used to identify or classify patterns.

Ibragimov et al. [34] used CNNs to segment the portal vein in liver CT images. Then, they used Markov random fields (MRF) to improve the results, which included removing isolated regions caused by segmentation errors. The results produced by CNN-MRF-based methods were supplemented with the detection of the PV centerline according to anatomical features of the PV such as its branch composition and tubularity.

The authors of [29] proposed automatic liver vessel segmentation based on a multi-pathway CNN architecture. This architecture combines a number of networks, where each network is to learn different features from the vessel image processed. The learning process was carried out for three planes (sagittal, coronal, and transverse) to fully extract the features.

Kehwani et al. [28] presented a multi-task 3D fully convolutional neural network (3D-FCN) for reconstructing the vessel tree. The proposed approach allows voxels to be detected on vascular centerlines and estimates the boundaries in the reconstructed vessel tree. A metric was defined that includes both inter-class distance and intra-class topological distance between vascular pairs. Vessel trees are then reconstructed using the learned connectivity metric and the shortest path tree algorithm.

In [31], the fuzzy connectedness (FC) method was employed for the three-dimensional segmentation of blood vessels of liver. A modified version of Jerman’s vesselness filter [79] was also used to amplify vessels in source images.

The accuracy of deep learning methods depends on the right selection of training data and the quantity of these data. Liver vessels are very complex and diverse. Unfortunately, the number of available datasets with annotations that can allow segmentation and 3D visualization is limited. What is more, significant discrepancies in the annotations of medical datasets, such as omissions of certain vessels, can be found due to the different clinical experience of annotators. The use of incorrect annotations causes segmentation errors in the supervised learning methods used. For this reason, it is worth trying deep learning methods using, e.g., the 3D U-Net architecture [80]. The 3D U-Net is a dense convolutional network that can perform volumetric segmentation using a small quantity of training data and incomplete annotations. Unfortunately, an unbalanced number of classes can often cause the training process to get stuck at the local minima of the loss function and ultimately produce a prediction error. Consequently, it is necessary to preliminarily calculate weighted parameters of the classes to compensate for their different frequency [80].

Huang et al. [30] chose the 3D-U-Net for extracting liver blood vessels, using several training samples and incomplete annotations. To improve segmentation results in unbalanced classes, the parameters are adjusted based on the number of correctly classified voxels of the foreground and the number of incorrectly classified voxels, based on the Dice loss function. At the same time, the penalty for incorrectly classified voxels is increased to teach the network to recognize vessels with weak boundaries, low contrast, and high noise.

The authors of [26] also employed a deep learning method utilizing the 3D U-Net architecture for the intraoperative segmentation of liver vasculature. Vessels were segmented based on ultrasound images. The 3D U-Net was used to train three different deep learning models for separate segmentation tasks: segmenting the entire vasculature and the hepatic and portal veins separately.

Mishra et al. [32] also segmented vessels in USG images, but they used CNNs. The CNN’s performance is affected by the quantity and complexity of the training data. Unfortunately, ultrasound images showing liver vessels are not generally available. In [32], USG images were divided into overlapping patches. Each patch was classified using the CNN as follows: fully or partially covers the region of the vessel (a positive vessel patch) or does not contain any part of the vessel region (negative non-vessel patch). Then, to perform pixel level segmentation, k-means unsupervised clustering was used with the best results.

Reference [25] presented a deep neural network employing an attention-guided concatenation (AGC) module and allowing an adaptive selection of context features from low-level features guided by high-level features to produce a detailed structure of the liver vessel. It is important that the segmentation extracts continuous liver vessels. Consequently, a multiscale feature fusion block was proposed as a functional block of the U-Net.

In the approach proposed in [27], first, a small number of vessels are annotated and taken to train a sparse dictionary and logistic regressor to allow producing an initial vessel classification. In the next step, a bootstrapping technique is used to train deep neural networks based on noisy liver vessel labels. In the test phase, post-processing based on predictions from the model is used to produce liver blood vessel trees.

Nazir et al. [24] proposed liver vessel segmentation by employing a cascade incremental learning (CIL) model. What is more, a ternary tree-based method to map all the possible liver vessel variants into their respective tree topologies was also used. The reason for using CIL for liver vessel segmentation is that it is difficult to provide a sufficient number of features to cover all variations in the liver vessel structure.

A summary of the analyzed supervised machine learning methods is presented in Table 7.

## 8. Challenges and Conclusions

Of the 31 articles included in the literature review:

- 21 studies presented methods using CT images [12,13,15,16,19,20,21,22,23,24,25,27,28,29,30,31,34,35,36,37,38]
- 8 papers concerned approaches that use CTA images [7,8,10,11,14,17,18,24]
- 2 articles presented methods that use MR images [13,33]
- 2 studies described approaches using USG images [26,32]
- 13 papers presented results of research based on publicly available datasets [12,13,15,16,24,25,27,28,29,30,31,37,38]
- 18 articles describe results obtained using private datasets [7,8,9,10,11,14,17,18,19,20,21,22,26,32,33,34,35,36]

The methods most commonly applied are machine learning ones. They account for a total of 15 articles. Unfortunately, it is difficult to clearly evaluate segmentation results because different datasets (i.e., public or private) were used. In addition, the reported metrics are not consistent. For the few publicly available datasets, there is no information about the experience and knowledge of experts who performed ground-truth segmentation. Obviously, manually segmenting complex liver vessels is very tedious and time-consuming and requires a large workload.

It can be said that image quality still has a very strong impact on segmentation results and that a method that is developed and works well for one dataset may turn out to be unsuitable for other image sets, e.g., containing a different level of noise or produced by other equipment. In addition, the authors of the research papers did not pay much attention to segmenting pathological vessels. The assumptions of a circular cross-section of vessels and their regular linearity will not lead to correct segmentation results if a pathology is present.

In some studies, the authors tried to present vessel segmentation methods for two imaging techniques: CT and MR [13], as well as CT and CTA [24], and this is a very valuable direction of research. However, the two sets analyzed in the publications [13,24] are not publicly available.

Liver vessel segmentation methods that use deep learning are becoming increasingly popular. This is due, among others, to the increasing computing power of computers. Moreover, deep learning allows the best-fit internal representation of the image to be extracted, while in classical machine learning algorithms, it is crucial to extract the right features of the image, which often requires specialist knowledge. The lack of sufficient public datasets led to another trend in deep learning methods. Unsupervised deep learning and semi-supervised learning are becoming more widespread [81,82,83], as are generative networks [84,85,86]. However, these approaches have not yet been used to segment liver vessels.

Recent years have seen an accelerating growth of medical image datasets made publicly available, but they are still limited to certain anatomical areas and also selected imaging methods. The availability of this data is increasing due to the spread of image diagnostics. Consequently, liver vessel segmentation methods that use deep learning will continue to develop as long as organizations publish large and labeled datasets created based on actual knowledge of appropriately experienced physicians. Of course, such datasets must include variations in vessel shape caused by individual characteristics of patients, as well as pathologies. The generalization of knowledge with the use of deep learning methods will certainly improve the ability to use the segmentation of liver blood vessels in practice.

For newly available datasets such as MSD [55] or CHAOS [56], a certain period of time must pass before advanced solutions enabling liver vessel segmentation appear. It is a very valuable initiative to organize competitions [87] as a part of which datasets and the results of experiments are made public and the appropriate metrics properly chosen by experts are used to evaluate segmentation results. This is a very good initiative, which would allow new, better methods to be developed and the results obtained to be standardized to some extent. However, there are still no publicly available USG datasets that would enable liver vessel segmentation. Because of the uneven background and low contrast, USG is an imaging technique in which segmenting liver vessels is the most difficult. Videos can also be recorded of the entire ultrasound examination. Real-time tracking and segmentation of moving objects is a subject well known in computer vision [88,89,90]. However, tracking selected organs during a USG examination (or in recorded videos) may be difficult, although publications on this subject can be found in the literature [91,92].

In order to develop repeatable, reliable, and traceable methods of segmenting liver vessels that could be useful in medical practice, experts in such fields as medicine, software engineering, and statistical analysis must closely cooperate with researchers who develop segmentation methods. In view of this requirement, none of the methods presented has overcome all the challenges found in liver vessel segmentation yet.

## Figures and Tables

**Table 1:** Summary of various imaging techniques and segmentation methods, based on the literature review. CT: computed tomography, CTA: computed tomography angiography, MR: magnetic resonance, USG: ultrasonography.

Author	Year	Imaging Technique	Segmentation Method
Zeng et al. [7]	2018	CTA	Active contour methods
Chung et al. [8]	2018	CTA	(Section 5)
Lu et al. [9]	2017	MR	
Cheng et al. [10]	2015	CTA	
Shang et al. [11]	2010	CTA	
Guo et al. [12]	2020	CT	Tracking methods
Lebre et al. [13]	2019	CT and MR	(Section 6)
Zeng et al. [14]	2018	CTA	
Sangsefidi et al. [15]	2018	CT	
Yang et al. [16]	2018	CT	
Zeng et al. [17]	2017	CTA	
Yan et al. [18]	2017	CTA	
Chi et al. [19]	2010	CT	
Bauer et al. [20]	2010	CT	
Alhonnoro et al. [21]	2010	CT	
Esneault et al. [22]	2009	CT	
Kaftan et al. [23]	2009	CT	
Nazir et al. [24]	2021	CT and CTA	Machine learning methods
Yan et al. [25]	2020	CT	(Section 7)
Thomson et al. [26]	2020	USG	
Xu et al. [27]	2020	CT	
Keshwani et al. [28]	2020	CT	
Kitrungrotsakul et al. [29]	2019	CT	
Huang et al. [30]	2018	CT	
Zhang et al. [31]	2018	CT	
Mishra et al. [32]	2018	USG	
Gocer et al. [33]	2017	MR	
Ibragimov et al. [34]	2017	CT	
Zeng et al. [35]	2016	CT	
Wang et al. [36]	2016	CT	
Oliveira et al. [37]	2011	CT	
Bruyninckx et al. [38]	2010	CT	

**Table 2:** Performance measures for liver vessel segmentation methods (*S*), in relation to the reference (*R*), based on thirty one studies in this review, where true positives (TP) are pixels classified correctly as positive, false positives (FP) are pixels classified incorrectly as positive, true negatives (TN) are pixels classified correctly as not positive, false negatives (FN) are pixels classified incorrectly as not positive. For contours marked *R* and *S*: *r* and *s* represent points belonging to the corresponding contours, while $d_{s}$ and $d_{r}$ are the distances from points *s* and *r* to the nearest points of the *R* and *S* contours.

Metrics	Standard Formula	Description
Sensitivity (Sens); recall; true positive rate (TPR) [42]	$TPR=Sens=\frac{TP}{TP+FN}$	Proportion of positives that are correctly identified.
Accuracy (Ac) [43]	$Ac=\frac{TP+TN}{TP+TN+FP+FN}$	Proportion of detected true samples that are actually true.
Specificity (Spec) [42]	$Spec=\frac{TN}{TN+FP}$	Proportion of negatives that are correctly identified.
Precision; positive predictive value (PPV) [44]	$PPV=\frac{TP}{TP+FP}$	Proportion of positive results that are true positives.
Negative predictive value (NPV) [44]	$NPV=\frac{TN}{TN+FP}$	Proportion of negative results that are true negatives.
False positive rate (FPR) [45]	FPR=1−Spec	Ratio of the number of negative samples wrongly categorized as positive (FP) to the total number of actual negative samples.
False negative rate (FNR) [45]	FNR=1−TPR	Ratio of the number of positive samples wrongly categorized as negative (FN) to the total number of actual positive samples.
Dice similarity coefficient (DSC) [46]	$DSC=\frac{2\cdot TP}{FP+FN+2\cdot TP}$	Similarity between two sample sets.
Jaccard similarity coefficient (JSC) [47]	$JSC=\frac{TP}{FP+FN+TP}$	Similarity between finite sample sets.
Volumetric overlap error (VOE) [48]	VOE=1−JSC	The VOE indicates segmentation performance; if the VOE is close to 0, this represents a perfect segmentation.
Distance error ($e_{D}$) [49]	$e_{D}=\frac{1}{\left|S\right|}\left(\sum_{s=1}^{\left|S\right|}\left|\;d_{s}\;\right|\right)$	Measure of the average distance calculated from all *s* points on *S* to the closest point on *R*.
Symmetric distance error ($e_{Dsym}$) [50]	$e_{Dsym}=\frac{1}{\left|S|+|R\right|}\left(\sum_{s=1}^{\left|S\right|}|\;d_{s}\;|+\sum_{r=1}^{\left|R\right|}\left|\;d_{r}\;\right|\right)$	Measure of the average distance calculated from all *s* points on *S* to the closest point on *R* and vice versa.
Root mean standard error (RMSE) [49]	$RMSE=\sqrt{\frac{1}{\left|S\right|}\left(\sum_{s=1}^{\left|S\right|}\left|\;d_{s}\;\right|\right)}$	Measure of the average squared difference between the estimated values and the actual value.
Root mean squared symmetric surface distance (RMSD) [51]	$RMSD=\sqrt{\frac{1}{\left|S|+|R\right|}}\times \sqrt{\sum_{x\in S}d^{2}\left(x,R\right)+\sum_{y\in R}d^{2}\left(y,S\right)}$	The RMSD indicates the segmentation performance between two contours *S* and *R*; the lower the RMSD, the better the segmentation result.
Hausdorff distance (HD) [52]	$HD=\max(\max_{s\in S}|\;d_{s}\;|,\;\max_{r\in R}|\;d_{r}\;|)$	Overlapping index, which measures the largest Euclidean distance between two contours *S* and *R* and vice versa, computed over all pixels of each curve.
Classification error (ϵ) [18]	$\epsilon =|B_{e}\left|/|B\right|$	Proportion of the incorrectly classified vessel branches $|B_{e}|$ to all vessel branches |B|.
Recognition rate (RR) [19]	$RR=|B_{t}\left|/|B\right|$	Proportion of the correctly classified vessel branches $|B_{t}|$ to all vessel branches |B|.

**Table 4:** Summary of active contour approaches for liver vessel segmentation (segmentation metrics are given in Table 2). CTA: computed tomography angiography, MR: magnetic resonance.

Author	Testing Dataset	Synthetic Data Used	Metrics Results
Zeng et al. [7]	12 CTA volumes	Yes	Ac=0.98,Sens=0.68,Spec=0.99,
			DSC=0.73,JSC=0.66,RMSD=2.56 mm
Chung et al. [8]	50 CTA images	No	DSC=0.96
Lu et al. [9]	5 MR volumes	No	TPR=0.81,FPR=0.02,DSC=0.81
Cheng et al. [10]	3 CTA datasets	Yes	JSC,Visual
Shang et al. [11]	20 CTA volumes	Yes	Visual

**Table 5:** Summary of tracking methods (segmentation metrics are given in Table 2). CT: computed tomography, CTA: computed tomography angiography, MR: magnetic resonance.

Author	Testing Dataset	Synthetic Data Used	Metrics Results
Guo et al. [12]	8 CT volumes (3D-IRCADb-01)	No	Ac=0.97,Sens=0.66,Spec=0.98
	(http://www.ircad.fr/research/3dircadb/)		
	(accessed on 5 May 2019)		
Lebre et al. [13]	20 CT (3D-IRCADb-01) volumes	Yes	Ac=0.97,Sens=0.69,Spec=0.98,
	(http://www.ircad.fr/research/3dircadb/)		PPV=0.61,FPR=0.01,FNR=0.32
	(accessed on 5 May 2019)		
	and 40 MR volumes from internal dataset		Ac=0.98,Sens=0.54,Spec=0.98
			PPV=0.3,FPR=0.01,FNR=0.45
Zeng et al. [14]	6 CTA volumes	Yes	Ac=0.98,Sens=0.8,Spec=0.99
Sangsefidi et al. [15]	50 CT volumes, including	Yes	Ac=0.93,Sens=0.93,Spec=0.93,
	20 CT (3D-IRCADb-01) volumes		DSC=0.93,JSC=0.88
	(http://www.ircad.fr/research/3dircadb/)		
	(accessed on 5 May 2019)		
Yang et al. [16]	10 CT datasets (SLIVER07 [51])	No	FP
Zeng et al. [17]	6 CTA volumes	Yes	Ac=0.98,Sens=0.8,Spec=0.99
Yan et al. [18]	6 CTA volumes	No	Ac=0.97,Sens=0.79,Spec=0.98,
			ϵ=1.5%
Chi et al. [19]	10 CT scans	No	Ac=0.98,RR=99%
Bauer et al. [20]	15 contrast enhanced CT	Yes	FN=0.26%
Alhonnoro et al. [21]	CT	No	RMSE
Esneault et al. [22]	CT	No	Visual
Kaftan et al. [23]	30 CT scans	No	Visual

**Table 6:** Summary of liver vessel segmentation approaches using unsupervised learning (segmentation metrics are given in Table 2). CT: computed tomography, MR: magnetic resonance.

Author	Testing Dataset	Synthetic Data Used	Metrics Results
Goceri et al. [33]	14 MR volumes	No	Ac,DSC,HD
Oliveira et al. [37]	15 CT datasets (SLIVER07 [51])	No	Visual
Zeng et al. [35]	6 CT volumes	No	Ac=0.98,Sens=0.74,Spec=0.99
Wang et al. [36]	18 CT volumes	No	$e_{D}=12.7$ mm
Bruyninckx et al. [38]	5 CT images (3D-IRCADb-01)	No	DSC
	(http://www.ircad.fr/research/3dircadb/)		
	(accessed on 5 May 2019)		

**Table 7:** Summary of liver vessel segmentation approaches using supervised learning (segmentation metrics are given in Table 2). CT: computed tomography, MR: magnetic resonance, USG: ultrasonography.

Author	Testing Dataset	Synthetic Data Used	Metrics Results
Ibragimov et al. [34]	72 CT images	No	$DSC=0.83,e_{Dsym}=1.08$ mm
Kitrungrotsakul et al. [29]	1 CT volume (3D-IRCADb-01)	Yes	Sens=0.9,DSC=0.92,PPV=0.84,
	(http://www.ircad.fr/research/3dircadb/)		VOE=17.2%
	(accessed on 5 May 2019)		
Keshwani et al. [28]	20 CT volumes from internal dataset	Yes	Sens=0.96,Spec=0.94,DSC=0.94
	and 20 CT (3D-IRCADb-01) volumes		Sens=0.96,Spec=0.91,DSC=0.92
	(http://www.ircad.fr/research/3dircadb/)		
	(accessed on 5 May 2019)		
Zhang et al. [31]	20 CT (3D-IRCADb-01) volumes	No	Ac=0.96,Sens=0.73,Spec=0.97,
	(http://www.ircad.fr/research/3dircadb/)		DSC=0.67
	(accessed on 5 May 2019)		
	20 CT (SLIVER07) datasets [51]		Ac=0.96,Sens=0.89,Spec=0.97,
			DSC=0.71
Huang et al. [30]	10 CT volumes from internal dataset and	Yes	Ac=0.97,Sens=0.76,Spec=0.98,
	20 CT (SLIVER07) datasets [51] and		DSC=0.75
	10 CT (3D-IRCADb-01) volumes		
	(http://www.ircad.fr/research/3dircadb/)		
	(accessed on 5 May 2019)		
Thomson et al. [26]	203 USG volumes	No	DSC=0.66
Mishra et al. [32]	132 USG images	No	JSC=0.69
Yan et al. [25]	10 CT volumes from internal dataset and	No	Sens=0.85,PPV=0.78,DSC=0.8
	20 CT (3D-IRCADb-01) volumes		Sens=0.93,PPV=0.99,DSC=0.9
	(http://www.ircad.fr/research/3dircadb/)		
	(accessed on 5 May 2019)		
Xu et al. [27]	20 CT (3D-IRCADb-01) volumes	No	Ac=0.99,Sens=0.78,Spec=0.99,
	(http://www.ircad.fr/research/3dircadb/)		DSC=0.68
	(accessed on 5 May 2019)		
Nazir et al. [24]	30 CTA internal datasets and	No	Ac= up to 98.90
	10 CT (SLIVER07) datasets [51]		Ac= up to 98.89

## Data Availability

No new data were created or analyzed in this study. Data sharing is not applicable to this article.

## Abbreviations

The following abbreviations are used in this manuscript:

3D	Tridimensional
Ac	Accuracy
ACM	Active contour model
AGC	Attention-guided concatenation
ANN	Artificial neural network
CIL	Cascade incremental learning
CNN	Convolutional neural network
CT	Computed tomography
CTA	Computed tomography angiography
DSC	Dice similarity coefficient
$e_{D}$	Distance error
$e_{Dsym}$	Symmetric distance error
ELM	Extreme learning machine
ϵ	Classification error
FC	Fuzzy Connectedness
FCM	Fully Convolutional neural network
FMM	Fast Marching method
FN	False negative
FNR	False negative rate
FP	False positive
FPR	False positive rate
HD	Hausdorff distance
HV	Hepatic vein
ITK	Insight Toolkit
JSC	Jaccard similarity coefficient
*M*	Metric tensor for minimum cost path
MCP	Minimum cost path
MR	Magnetic resonance
MRF	Markov random fields
NPV	Negative predictive value
PPV	Positive predictive value
PV	Portal vein
R	Reference
RMSE	Root mean standard error
RANSAC	Random sample consensus
RMSD	Root mean square symmetric surface distance
RR	Recognition rate
S	Segmentation
Sens	Sensitivity
Spec	Specificity
TP	True positive
TN	True negative
USG	Ultrasonography
VOE	Volumetric overlap error
